# Diabetic Neuropathy: Prevalence and Impact on Quality of Life in Al-Ahsa, Saudi Arabia

**DOI:** 10.7759/cureus.33176

**Published:** 2022-12-31

**Authors:** Arwa M Alhajji, Zainab K Alkhlaif, Sarah A Bukhamsin, Fatimah S Alkhars, Hessah Al-Hussaini

**Affiliations:** 1 College of Medicine, King Faisal University, Al-Ahsa, SAU; 2 Endocrinology and Diabetes, King Faisal University, Al-Ahsa, SAU

**Keywords:** al-ahsa, saudi arabia, diabetic neuropathy, quality of life, diabetes mellitus

## Abstract

Objective

This study aims to measure the prevalence of diabetic neuropathy (DN) in patients with type 1 and type 2 diabetes mellitus (DM) and to explore the impact of DN on quality of life (QoL) in type 1 and type 2 DM patients in Al-Ahsa, Saudi Arabia.

Methods

This cross-sectional study targeted type 1 and type 2 DM patients who live in Al-Ahsa, Saudi Arabia. Self-reported online questionnaires distributed randomly on social media were used. The survey included three parts: sociodemographic data, the Self-Administered Leeds Assessment of Neuropathic Symptoms and Signs (S-LANSS) questionnaire, and the modified Arabic Diabetes Quality of Life (DQoL) questionnaire. The data have been collected from April 2022 to May 2022.

Results

The study included participants (n = 329) of both type 1 and type 2 DM. Patients' age ranged from 18 to 82 years with a mean age of 45.9 ± 15.2 years. A total of 166 (50.5%) patients were males and 319 (97%) were Saudi nationals. The prevalence of DN in the study population was 44.1%. Of the patients with DN, 73.1% have low QoL, which means DN increased the risk of low QoL by about four times (OR = 3.9; 95% CI: 2.5-6.3).

Conclusion

In conclusion, the study showed that the prevalence of DN in Al-Ahsa, Saudi Arabia was 44.1%. The presence of DN was associated with reduced QoL. Type 2 DM, low educational level, and the presence of other comorbidities were significantly associated with low QoL.

## Introduction

Diabetes mellitus (DM) is a group of metabolic diseases characterized by hyperglycemia resulting from defects in insulin secretion, insulin action, or both. It is further classified as type 1, type 2, gestational, and other specific types [1]. Type 1 and type 2 DM are the chief subclassifications. Commonly in adults, type 2 DM is observed. Patients with type 2 DM are resistant to insulin or do not make enough insulin. Type 1 DM, also known as insulin-dependent diabetes, is characterized by the absence or low levels of insulin due to the destruction of beta cells in the pancreas, typically secondary to an autoimmune process [2]. In Saudi Arabia, a study reported by the Ministry of Health described a rise from 0.9 million DM patients in 1992 to 2.5 million DM patients in 2010. Two factors have been associated with the great rise: the rising rate of obesity and the aging population [3].

DM is the leading cause of several complications worldwide. The most common complication is diabetic neuropathy (DN). DN is defined as the presence of symptoms and/or signs of peripheral nerve dysfunction in people with diabetes after the exclusion of other causes [4]. It is also characterized by pain and significant morbidity. Over time, at least 50% of diabetic patients develop DN. Glucose control effectively impedes the progression of DN in patients with type 1 diabetes, but the effect is weaker in patients with type 2 diabetes, which are considered the majority [5,6]. In Saudi Arabia, the prevalence of DN varied according to the studied regions. A hospital-based study conducted in Riyadh revealed that 69.2% of type 2 DM participants have peripheral DN. Another prospective study conducted in King Abdulaziz University Hospital in Jeddah that studied 237 patients with DM revealed that 56% have symptomatic peripheral DN. A study done in Qassim revealed that 38.2% of patients have DN [7]. Many factors are associated with the progression of diabetes to DN such as the duration of diabetes, poor glycemic control and poor compliance with treatment, low level of education, having comorbidities, smoking, and obesity [6,7]. The association of the risk factors to DN ascertains the importance of early recognition and proper risk-modifying interventions [8]. As was already mentioned, there is an increase in the prevalence of DM in Saudi Arabia, and as a result, complications are also expected to be high. And because there is no single study in Al-Ahsa that evaluates the prevalence of DN, our study aims to measure the prevalence of DN in patients with type 1 and type 2 DM, and to explore the impact of DN on quality of life (QoL) in diabetic patients in Al-Ahsa, Saudi Arabia.

## Materials and methods

Participants

The subjects for this survey were patients with type 1 and type 2 DM who live in Al-Ahsa, Saudi Arabia. A total of 394 patients responded to the online questionnaire, and 329 met the eligibility criteria. The eligibility criteria were as follows: (1) providing written consent; (2) having type 1 or type 2 DM; (3) aged 18 years and above; and (4) living in Al-Ahsa, Saudi Arabia.

Procedure

This was a cross-sectional study, where self-reported online questionnaires were distributed randomly on social media (WhatsApp and Telegram). The study was conducted from 15 April to 31 May 2022.

Measures

All data were retrieved from self-reported questionnaires. The first part of the questionnaire included patient characteristics. The following data were obtained: (1) patient’s sociodemographic data (age, gender, nationality, marital status, level of education, income, BMI, and province); (2) brief diabetic history (type of DM, duration of DM, type, and the number of medications, last glycosylated hemoglobin (HbA1c) result, and compliance to medication); and (3) the presence of comorbidities and smoking history.

The second part of the questionnaire included the Self-Administered Leeds Assessment of Neuropathic Symptoms and Signs (S-LANSS) questionnaire, which was used to assess DN. A score of 12 or more out of 24 suggests pain of predominantly neuropathic origin.

The third part included the modified Arabic Diabetes Quality of Life (DQoL) questionnaire, which assesses participants’ QoL. The questionnaire consists of three scales: satisfaction, the impact of diabetes, and worries about diabetes. DQoL profile is considered to be low when the mean score is higher than the population means.

Ethical issues

Participants were informed about the goal of the study and written consent was obtained. All data were compiled in a spreadsheet anonymously and confidentially under the primary investigator's responsibility. Ethical approval was granted by the Research Ethics Committee at King Faisal University via reference number KFU-REC-2022-FEB-EA000414 (dated: 01/02/2022).

Data analysis

The data were collected, reviewed, and then fed to Statistical Package for the Social Sciences (SPSS) version 21 (IBM Corp., Armonk, NY). All statistical methods used were two-tailed with an alpha level of 0.05, considering significance if the p-value is less than or equal to 0.05. The categorical data were given as numbers (n) and percentages (%), while mean and standard deviation (SD) were calculated for the continuous variables. Secondly, the association of sociodemographic and clinical factors with DQoL was assessed using the chi-square test and exact probability test for small frequency distributions. The exact regression model and odds ratio were approved to assess the effect of DN on QoL.

## Results

A total of 329 diabetic patients fulfilling the inclusion criteria were included. Patients' age ranged from 18 to 82 years with a mean age of 45.9 ± 15.2 years. A total of 166 (50.5%) patients were males and 319 (97%) were Saudi nationals. A total of 218 (66.3%) were married and 167 (50.8%) were university graduates. As for smoking, 73 (22.2%) were smokers and 199 (60.5%) were non-smokers. A total of 120 (36.5%) had normal body weight, 108 (32.8%) had overweight, and 101 (30.7%) were obese (Table 1).

**Table 1:** Sociodemographic and patient characteristics

Sociodemographic data	Number	%
Age in years		
<30	65	19.8%
30-49	117	35.6%
50-60	99	30.1%
>60	48	14.6%
Gender		
Male	166	50.5%
Female	163	49.5%
Nationality		
Saudi	319	97.0%
Non-Saudi	10	3.0%
Marital status		
Single	56	17.0%
Married	218	66.3%
Divorced/widow	55	16.7%
Educational level		
Below secondary	65	19.8%
Secondary	97	29.5%
University/above	167	50.8%
Income		
<5,000 Saudi riyal (SR)	80	24.3%
5,000-14,000 SR	111	33.7%
15,000-24,000 SR	88	26.7%
25,000+ SR	50	15.2%
Smoking		
Current smoker	73	22.2%
Ex-smoker	57	17.3%
Non-smoker	199	60.5%
Body mass index		
Normal weight	120	36.5%
Overweight	108	32.8%
Obese	101	30.7%

Brief diabetic history of the study population is shown in Table 2. A total of 204 (62%) patients had type 2 DM, while 60 (18.2%) had type 1 DM. A total of 122 (37.1%) patients were diagnosed with DM for less than five years and 79 (24%) for more than 15 years. HbA1c was 8 or more among 49.9% of the patients. A total of 173 (52.6%) patients were on oral hypoglycemic drugs (OHDs), while 52 (15.8%) were on insulin and 104 (31.6%) were on both. A total of 229 (69.6%) patients reported being usually compliant with the treatment while 87 (26.4%) were compliant only for some time and 13 (4%) were not compliant. A total of 141 (42.9%) patients had other comorbidities.

**Table 2:** Diabetes clinical data DM: diabetes mellitus; HbA1c: glycosylated hemoglobin; OHDs: oral hypoglycemic drugs.

Diabetes data	Number	%
Type of DM		
Type 1 DM	60	18.2%
Type 2 DM	204	62.0%
I don't know	65	19.8%
Duration of DM		
<5 years	122	37.1%
5-10 years	87	26.4%
11-15 years	41	12.5%
>15 years	79	24.0%
HbA1c		
<7	76	23.1%
7-7.9	89	27.1%
8-8.9	70	21.3%
9-9.9	26	7.9%
10+	13	4.0%
I don't know	55	16.7%
Received treatment		
OHDs	173	52.6%
Insulin	52	15.8%
Both of them	104	31.6%
If OHDs, how many		
1 type	126	45.5%
2 types	98	35.4%
3 types/more	53	19.1%
Regular treatment intake		
Usually	229	69.6%
Sometimes	87	26.4%
No	13	4.0%
Other comorbidities		
Yes	141	42.9%
No	188	57.1%

**Figure 1:**
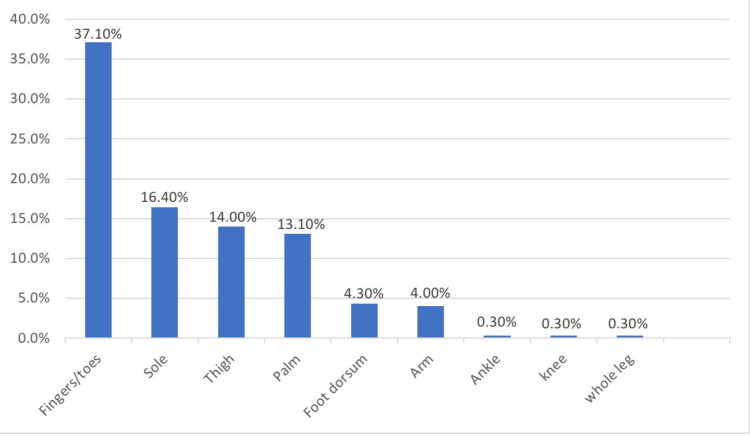
Frequency of site of pain

The most commonly reported site of pain in diabetic patients in the study was toes/fingers (37.1%), followed by sole (16.4%), thigh (14.0%), and palm (13.1%), as shown in Figure 1.

**Figure 2:**
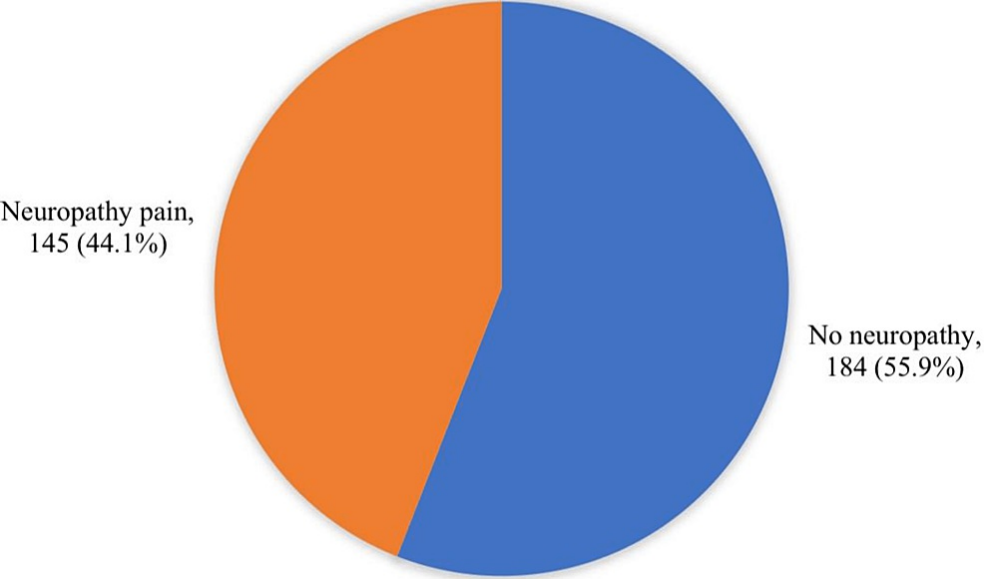
Prevalence of diabetic neuropathy

The results of the S-LANSS pain score are shown in Table 3. A total of 63.5% of the patients feel pins and needles, numbness, or tingling sensation at the pain site. Of the patients, 47.1% reported that pain comes on suddenly and in episodes for no apparent reason when they are completely calm, 44.4% reported that in the painful area, their skin feels unusually hot, like a burning pain, and 42.2% feel comfortable after massage in the pain area while 35.6% feel a loss of sensation/hypoesthesia on pressing the pain area. The overall mean score was 10.6 out of 24 (44.2%), and the prevalence of DN among the study population was 145 (44.1%) (Figure 2).

**Table 3:** Neuropathic pain frequency

Neuropathic pain items	Number	%
In the pain area, do you also feel pins and needles, numbness, or tingling sensation?		
Not at all	120	36.5%
Yes, usually	209	63.5%
Does your pain change the color of the skin in the painful area so that it looks different from normal, such as appearing speckled or redder when the pain is intense?		
No	216	65.7%
Yes	113	34.3%
Does your pain make the affected area abnormally sensitive to touch, such as feeling pain when lightly hitting the skin?		
No	202	61.4%
Yes	127	38.6%
Does your pain come on suddenly and in episodes for no apparent reason when you are completely calm, such as feeling an electric shock, jumping, or exploding?		
No	174	52.9%
Yes	155	47.1%
In the painful area, does your skin feel unusually hot, like a burning pain?		
No	183	55.6%
Yes	146	44.4%
How does the massage feel in the painful place?		
Feeling no difference	190	57.8%
I feel uncomfortable	139	42.2%
How does the pressure feel in the painful place?		
Feeling no difference	212	64.4%
Feel the loss of sensation/hypoesthesia	117	35.6%
Mean ± SD, %	10.6 ± 7.8, 44.2%	

Neuropathy pain was detected among 30.8% of patients aged less than 30 years versus 58.3% of others aged more than 60 years (P = 0.031). Also, 30.4% of single patients had neuropathy pain compared to 56.4% of the divorced group (P = 0.022). Neuropathy pain was detected among 61.5% of low-educated patients versus 37.7% of university graduates (P = 0.005). Additionally, 66.7% of ex-smokers had neuropathy pain compared to 36.7% of non-smokers (P = 0.001). A total of 47.1% of patients with type 2 DM had neuropathy pain versus 28.3% of type 1 diabetics (P = 0.036). Also, 66.3% of those who use insulin and oral medication had neuropathy pain compared to 33.5% of others on insulin only (P = 0.001). Additionally, 60.3% of patients with other comorbidities had neuropathy pain versus 31.9% of others without comorbidities (P = 0.001) (Table 4).

**Table 4:** Factors associated with diabetic neuropathy P: Pearson X2 test; $: exact probability test; * P < 0.05 (significant). DM: diabetes mellitus; SR: Saudi riyal; OHDs: oral hypoglycemic drugs.

Factors		Pain nature				P-value
		No neuropathy		Neuropathy pain		
		Number	%	Number	%	
Age in years	<30	45	69.2%	20	30.8%	0.031*
	30-49	66	56.4%	51	43.6%	
	50-60	53	53.5%	46	46.5%	
	>60	20	41.7%	28	58.3%	
Gender	Male	92	55.4%	74	44.6%	0.852
	Female	92	56.4%	71	43.6%	
Marital status	Single	39	69.6%	17	30.4%	0.022*
	Married	121	55.5%	97	44.5%	
	Divorced/widow	24	43.6%	31	56.4%	
Educational level	Below secondary	25	38.5%	40	61.5%	0.005*
	Secondary	55	56.7%	42	43.3%	
	University/above	104	62.3%	63	37.7%	
Income	<5,000 SR	52	65.0%	28	35.0%	0.130
	5,000-14,000 SR	56	50.5%	55	49.5%	
	150,00-24,000 SR	52	59.1%	36	40.9%	
	25,000+ SR	24	48.0%	26	52.0%	
Smoking	Current smoker	39	53.4%	34	46.6%	0.001*
	Ex-smoker	19	33.3%	38	66.7%	
	Non-smoker	126	63.3%	73	36.7%	
Body mass index	Normal weight	73	60.8%	47	39.2%	0.303
	Overweight	60	55.6%	48	44.4%	
	Obese	51	50.5%	50	49.5%	
Duration of DM	<5 years	76	62.3%	46	37.7%	0.268
	5-10 years	46	52.9%	41	47.1%	
	11-15 years	19	46.3%	22	53.7%	
	>15 years	43	54.4%	36	45.6%	
Type of DM	Type 1 DM	43	71.7%	17	28.3%	0.036*^$^
	Type 2 DM	108	52.9%	96	47.1%	
	I don't know	33	51.6%	32	48.4%	
Received treatment	OHDs	115	66.5%	58	33.5%	0.001*
	Insulin	34	65.4%	18	34.6%	
	Both of them	35	33.7%	69	66.3%	
Regular treatment intake	Usually	141	61.6%	88	38.4%	0.003*
	Sometimes	35	40.2%	52	59.8%	
	No	8	61.5%	5	38.5%	
Other comorbidities	Yes	56	39.7%	85	60.3%	0.001*
	No	128	68.1%	60	31.9%	

The mean of DM patients’ overall DQoL score was 75.2 ± 20.8, with 55% of participants having low QoL. The overall mean satisfaction score was 35.1 out of 70, where 156 (47.4%) had an overall satisfaction score below the mean, indicating a low satisfaction level (Table 5). As for the impact of DM on patients’ life, the mean score was 29.1 ± 9.2 out of 55, where 160 (48.6%) had a high impact of DM, which indicates low QoL. The third scale in the DQoL questionnaire was diabetes-related worry, which scored 11.1 ± 3.8 out of 20, as 163 (49.5%) had high worry levels (Table 6).

**Table 5:** Diabetes quality of life (life satisfaction)

Satisfaction items	Very satisfied		Moderately satisfied		Neither satisfied nor dissatisfied		Moderately dissatisfied		Very dissatisfied	
	Number	%	Number	%	Number	%	Number	%	Number	%
Time takes to manage diabetes	109	33.1%	75	22.8%	104	31.6%	31	9.4%	10	3.0%
Time spent getting checkups	86	26.1%	74	22.5%	128	38.9%	32	9.7%	9	2.7%
The time it takes to determine the sugar level	99	30.1%	65	19.8%	127	38.6%	26	7.9%	12	3.6%
Current treatment	86	26.1%	68	20.7%	129	39.2%	36	10.9%	10	3.0%
The flexibility you have in your diet	65	19.8%	86	26.1%	118	35.9%	43	13.1%	17	5.2%
Burden your diabetes is placing on your family	58	17.6%	79	24.0%	138	41.9%	43	13.1%	11	3.3%
Knowledge about your diabetes	62	18.8%	74	22.5%	149	45.3%	35	10.6%	9	2.7%
Sleep	59	17.9%	82	24.9%	135	41.0%	34	10.3%	19	5.8%
Social relationships and friendships	91	27.7%	69	21.0%	126	38.3%	33	10.0%	10	3.0%
Work, school, and household activities	79	24.0%	72	21.9%	128	38.9%	40	12.2%	10	3.0%
The appearance of your body	62	18.8%	74	22.5%	137	41.6%	37	11.2%	19	5.8%
The time you spend exercising	42	12.8%	81	24.6%	119	36.2%	51	15.5%	36	10.9%
Your leisure time	73	22.2%	78	23.7%	137	41.6%	26	7.9%	15	4.6%
Life in general	87	26.4%	74	22.5%	129	39.2%	29	8.8%	10	3.0%
Mean ± SD	35.1 ± 11.9									
% of patients < mean (low satisfaction)	47.4%									

**Table 6:** Diabetes quality of life (impact and worries) QoL: quality of life.

	Never		Sometimes		Often		Frequently		Always	
	Number	%	Number	%	Number	%	Number	%	Number	%
Impact items										
Feel pain associated with the treatment	71	21.6%	68	20.7%	128	38.9%	49	14.9%	13	4.0%
Embarrassed by having to deal with your diabetes in public	106	32.2%	53	16.1%	111	33.7%	46	14.0%	13	4.0%
Have low blood sugar	48	14.6%	86	26.1%	131	39.8%	44	13.4%	20	6.1%
Feel physically ill	42	12.8%	79	24.0%	133	40.4%	55	16.7%	20	6.1%
Feel good about yourself	45	13.7%	46	14.0%	121	36.8%	85	25.8%	32	9.7%
Interfere with driving a car or using a machine	110	33.4%	56	17.0%	101	30.7%	44	13.4%	18	5.5%
Times for explaining what it means to have diabetes	49	14.9%	99	30.1%	126	38.3%	39	11.9%	16	4.9%
Should tell others about your diabetes	34	10.3%	94	28.6%	135	41.0%	50	15.2%	16	4.9%
Teased because you have diabetes	95	28.9%	66	20.1%	110	33.4%	43	13.1%	15	4.6%
Go to the bathroom more than others due to diabetes	32	9.7%	83	25.2%	148	45.0%	53	16.1%	13	4.0%
Eat something you should not rather than tell someone that you have diabetes	74	22.5%	72	21.9%	127	38.6%	40	12.2%	16	4.9%
Mean ± SD	29.1 ± 9.2									
% of patients > mean (high impact)	48.6%									
Worry items										
Worry whether you will be able to take a vacation	87	26.4%	60	18.2%	124	37.7%	39	11.9%	19	5.8%
Worry about whether you will pass out	69	21.0%	68	20.7%	130	39.5%	39	11.9%	23	7.0%
Worry that your body looks different because you have diabetes	63	19.1%	57	17.3%	130	39.5%	49	14.9%	30	9.1%
Worry that you will get complications from your diabetes	30	9.1%	41	12.5%	158	48.0%	59	17.9%	41	12.5%
Mean ± SD	11.1 ± 3.8									
% of patients > mean (high worry)	49.5%									
Total quality of life score (out of 65 total points)	75.2 ± 20.8									
% patients > mean (low QoL)	55%									

A total of 48.5% of highly educated patients had good QoL versus 32.3% of low-educated patients (P = 0.048). Also, 61.7% of patients with type 1 DM had good QoL compared to 44.1% of others with type 2 DM (P = 0.004). Good QoL was detected among 58.4% of patients receiving OHDs versus 17.3% of others who had OHDs and insulin (P = 0.001). Additionally, 50.2% of patients who were compliant with their treatment had good QoL versus 30.8% of others who did not (P = 0.015). Also, 50% of patients with no other comorbidities had good QoL in comparison to 38.3% of those with comorbidities (P = 0.035) (Table 7).

**Table 7:** Factors associated with quality of life P: Pearson X2 test; $: exact probability test; * P < 0.05 (significant). DM: diabetes mellitus; OHDs: oral hypoglycemic drugs; QoL: quality of life; SR: Saudi riyal.

Factors		The overall quality of life (QoL)				P-value
		Low QoL		Good QoL		
		Number	%	Number	%	
Age in years	<30	41	63.1%	24	36.9%	0.288
	30-49	58	49.6%	59	50.4%	
	50-60	53	53.5%	46	46.5%	
	>60	29	60.4%	19	39.6%	
Gender	Male	87	52.4%	79	47.6%	0.338
	Female	94	57.7%	69	42.3%	
Marital status	Single	29	51.8%	27	48.2%	0.795
	Married	120	55.0%	98	45.0%	
	Divorced/widow	32	58.2%	23	41.8%	
Educational level	Below secondary	44	67.7%	21	32.3%	0.048*
	Secondary	51	52.6%	46	47.4%	
	University/above	86	51.5%	81	48.5%	
Income	<5,000 SR	43	53.8%	37	46.3%	0.918
	5,000-14,000 SR	59	53.2%	52	46.8%	
	15,000-24,000 SR	50	56.8%	38	43.2%	
	25,000+ SR	29	58.0%	21	42.0%	
Smoking	Current smoker	37	50.7%	36	49.3%	0.069
	Ex-smoker	44	77.2%	13	22.8%	
	Non-smoker	100	50.3%	99	49.7%	
Body mass index	Normal weight	72	60.0%	48	40.0%	0.082
	Overweight	50	46.3%	58	53.7%	
	Obese	59	58.4%	42	41.6%	
Duration of DM	<5 years	64	52.5%	58	47.5%	0.785
	5-10 years	47	54.0%	40	46.0%	
	11-15 years	25	61.0%	16	39.0%	
	>15 years	45	57.0%	34	43.0%	
Type of DM	Type 1 DM	23	38.3%	37	61.7%	0.004*
	Type 2 DM	114	55.9%	90	44.1%	
	I don't know	44	67.7%	21	32.3%	
Received treatment	OHDs	72	41.6%	101	58.4%	0.001*
	Insulin	23	44.2%	29	55.8%	
	Both of them	86	82.7%	18	17.3%	
Compliance to treatment	Usually	114	49.8%	115	50.2%	0.015*
	Sometimes	58	66.7%	29	33.3%	
	No	9	69.2%	4	30.8%	
Other comorbidities	Yes	87	61.7%	54	38.3%	0.035*
	No	94	50.0%	94	50.0%	

A total of 59.2% of patients without DN had good QoL versus 26.9% of others with neuropathy, as DN increased the risk for having low QoL by about four times (OR = 3.9; 95% CI: 2.5-6.3) (Table 8).

**Table 8:** Effect of diabetic neuropathy on quality of life * P < 0.05 (significant).

Pain nature	The overall quality of life (QoL)				P-value	OR (95% CI)
	Low QoL		Good QoL			
	Number	%	Number	%		
No neuropathy	75	40.8%	109	59.2%	0.001*	1
Neuropathy pain	106	73.1%	39	26.9%		3.9 (2.5-6.3)

## Discussion

DN is defined as a “heterogeneous group of conditions that affect different parts of the nervous system and present with diverse clinical manifestations.” The commonest form of DN is distal symmetrical polyneuropathy, which accounts for 75% of DN [9,10]. The estimated prevalence of peripheral DN among adults with diabetes in the US is 19.4% (15.5-23.2%), and 26.2% (18.9-33.5%) among persons with diabetes for more than 10 years [11]. DN is associated with a number of complications, mainly diabetic foot syndrome, which is the main reason for hospitalization [10]. The presence of DN doubles the hazard of limb amputation compared with DM patients without neuropathy; the presence of a foot deformity, as a consequence of muscle wasting, also a result of motor neuropathy, upsurges the risk of amputations 12-fold, and if the patient is diagnosed with a diabetic foot ulcer, the risk is 36-fold higher [12].

The current study aimed to measure the prevalence of DN and its impact on QoL in Al-Ahsa, Saudi Arabia. The study revealed that less than half of the patients (44.1%) had neuropathic pain, of whom 47.1% are type 2 diabetics, and 28.3% of them are type 1 diabetics. Andrei Cristian et al. [13] estimated that about 28.70% of patients with type 1 DM and 50.70% of patients with type 2 DM had neuropathy. These results are similar to our study findings. Another study assessed the prevalence of type 1 DM complications in many European countries and found that the prevalence of DN was about 28% [14], which is much lower than the frequency of DN identified in our study patients. In the UK, a study reported that the prevalence of DN in type 2 DM was 32.1%, and in patients over 60 years old, the prevalence exceeded 50% [15]. Yovera-Aldana et al. [16] conducted a systematic review and concluded that the estimated pooled prevalence of DN in Latin America and the Caribbean was 46.5% (95% CI: 38%-55%). In Al Madinah, Saudi Arabia, Sendi et al. [17] estimated the prevalence of DN at 30.1% in type 2 diabetic patients and 25.9% in type 1 diabetic patients, with an overall prevalence of 29.1%, which is lower than the current estimated prevalence. On the other hand, a prevalence of 65.3% was detected for painful peripheral DN in a nationally representative diabetic population in 2010. Some possible explanations for the variation in these study findings are different diagnostic criteria implicated and different study populations. Peripheral DN was significantly higher in old age, high duration of DM, uncontrolled HbA1c, and positive family history of DM [18]. In other Middle Eastern countries, the prevalence of painful peripheral DN was 61.3% for Egyptian diabetic patients, 57.5% for Jordanian, 53.9% for Lebanese, and 37.1% for the population in the Gulf States [19].

As for patients' QoL, the current study showed that more than half of DM patients had poor QoL. In regards to patients' satisfaction, patients were dissatisfied the most regarding the time they spend exercising (26.4%). Regarding the impact of DM on QoL, 45% of patients mentioned that they go to the bathroom more than others, while 40.4% reported that they often feel physically ill. For the worry domain, the results showed that the most worrying thing for the patients was that their body looks different because of diabetes. Other studies show that the most frequently reported difficulties due to DM and the ones which have a major effect on patients’ QoL were pain/discomfort (68.0%), followed by mobility restriction, depression, and anxiety. Also, other studies that assessed the effect of DM on patients’ QoL showed consistent findings that the disease has an undesirable influence on their daily life due to its adverse effects [20-22]. Furthermore, the negative impact on QoL is higher if diabetic patients experienced complications [23-25]. This is mainly caused by increasing physical discomfort, decreasing activity, and reducing their physical state [26].

The study also revealed that more than half (59.2%) of the patients without DN had good QoL versus only one-fourth of others with neuropathy, as DN increased the risk for having low QoL by about four times (OR = 3.9; 95% CI: 2.5-6.3). This was consistent with the findings of many other studies [27-30].

Finally, this is a cross-sectional study, which used an online-distributed questionnaire to collect the data. This may result in recall bias, so further studies with hospital-based records and interviews are recommended.

## Conclusions

In conclusion, our study showed that the prevalence of DN was 44.1%. A total of 73.1% of patients with neuropathic pain have low QoL, as the presence of DN was associated with reduced QoL by about four times. Type 2 DM and the presence of other comorbidities were significantly associated with low QoL. Proper management of diabetes is crucial to prevent or delay DN, therefore, improving patients’ QoL.
